# Serotype Distribution of *Aggregatibacter actinomycetemcomitans* in Periodontitis Patients

**DOI:** 10.3390/pathogens14080805

**Published:** 2025-08-13

**Authors:** Nabil Khzam, Omar Kujan, Dorte Haubek, Leticia Algarves Miranda

**Affiliations:** 1 Dental School, The University of Western Australia, Nedlands, WA 6009, Australia; omar.kujan@uwa.edu.au (O.K.); leticia.algarvesmiranda@uwa.edu.au (L.A.M.); 2 NK Periodontics, Specialist Periodontal Private Practice, Applecross, WA 6155, Australia; 3 Jammerbugt Municipal Dental Service, DK-9460 Brovst, Denmark; dorte.haubek@outlook.dk

**Keywords:** *Aggregatibacter actinomycetemcomitans*, serotypes, periodontitis

## Abstract

**Aim:** The aim of the current investigation was to detect serotypes of *Aggregatibacter actinomycetemcomitans* in a cohort of Western Australians diagnosed with periodontitis. **Materials and Methods:** A total of 64 subjects were selected. Intra-oral samples were taken from every subject in the present investigation. Periodontal, radiographical, and microbiological analyses were conducted. A polymerase chain reaction was employed to investigate the presence of *Aggregatibacter actinomycetemcomitans* serotypes. **Results:** Only twelve (18.75%) patients were tested positive for *Aggregatibacter actinomycetemcomitans*. The most dominant serotypes of *Aggregatibacter actinomycetemcomitans* in this group were serotype e (80.55%), followed by serotype c (52.77%). Both serotypes b and d were absent in the present investigation. Serotype e presented in isolation or combined with other serotypes. The other serotypes tend to be present alone, but when they were isolated together, they were always combined with serotype e. It seems that serotype e of *Aggregatibacter actinomycetemcomitans* is associated with those who live in rural areas (*p* = 0.003), and those with low education (*p* = 0.041), and severe forms of periodontitis in this cohort. **Conclusions:** In patients diagnosed with severe periodontitis, serotype e was dominant in this population. Serotypes b and d did not appear in the present study.

## 1. Introduction

Periodontitis is characterized by microbially initiated and host-mediated chronic inflammation that results in the loss of periodontal attachment and alveolar bone support around the teeth [1]. Periodontitis has always been presented as an oral disease affecting the periodontal tissue [2]. However, periodontitis is able to influence the overall health of patients and their mental health. which can be a characteristic of some systemic diseases [3]. Periodontitis is a common systemic disease and is considered to be one of the most important causative factors of tooth loss and the increased risk of systemic diseases. The complex and multicausal inflammatory response in periodontitis is initiated by an interaction between genetics, age, lifestyle, and the accumulation of dental plaque in the periodontal region [4]. *A. actinomycetemcomitans* is a Gram-negative facultative anaerobic, an early invader, which belongs to the *Pasteurellaceae* family [5]. *A. actinomycetemcomitans* was reported in 1912 in human actinomycosis infection and was called *Bacterium actinomycetem comitans* [6]. *A. actinomycetemcomitans* and its implication in the etiology of periodontitis was captured after the isolation and the discovery of leukotoxin [7].

In 1983, Zambon and coauthors were the first to discover the three distinct surface antigens on the surface of the *Actinobacillus actinomycetemcomitans* with the discovery of serotype b which is considered responsible for the pathogenicity of this bacterium [8]. *A. actinomycetemcomitans* has two genotypes namely, JP2 and non-JP2, and seven serotypes, a to g [9,10,11]. Each one of those serotypes represents a distinct clonal lineage of *A. actinomycetemcomitans*. Bacterial serotypes are frequently associated with disease. Different virulence factors can trigger an anti-inflammatory reaction in the host immune response, which can also be activated by certain distinct groups of strains from one single species, such as *Haemophilus influenzae* [12]. Specific serotypes have the ability to increase the virulence of a microbe, and the case of *Neisseria Meningitidis* is an example of meningococcal disease [13]. In periodontics, microbes like *Porphyromonas gingivalis* and *A. actinomycetemcomitans* have a number of serotypes that can enhance their virulence [8,14].

The distribution of these serotypes varies according to the patient’s ethnicity and geographic location [15]. Serotype b is a common finding in those patients with severe periodontitis [8]. This is particularly the case for the virulent clonal lineage of *A. actinomycetemcomitans* serotype b, termed the JP2 genotype, which is characterized by a 530 base pair deletion in the promoter region of the leukotoxin apron; this genotype was originally isolated from patients with African descent [16,17]. The JP2 genotype of *A. actinomycetemcomitans* is 10 to 12 times more responsible for the release of leukotoxins than the non-JP2 genotype. The JP2 genotype of *A. actinomycetemcomitans* is associated with aggressive periodontitis [17]. Strains a, b, and c have a common presentation worldwide [18]. In the Japanese population with periodontitis, there is a high level of serotype e [19]. In the Middle East (Turkey) and South America (Brazil), serotypes a and c seem to have a high presence, especially in patients with early-onset periodontitis [20]. The US has one predominant serotype, b, that seems to be higher than other serotypes [21]. Serotype c seems to be very common in Asian countries like Korea, China, Japan, and Vietnam [22,23,24,25]. In North European countries, the three serotypes a, b, and c are very common in occurrence [26]. In Oceania, no single study has explored the presence of *A. actinomycetemcomitans* serotypes; hence, the purpose of this investigation is to detect serotypes in populations that live in Perth City in Western Australia.

## 2. Materials and Methods

### 2.1. Study Characterictics

A cross-sectional descriptive study of sixty-four consecutive patients with periodontitis, aged 40 years and younger, was conducted, with recruitment in two private specialist periodontal practices in Perth city, Western Australia, between March and June 2024. The inclusion criteria of the present investigation included Western Australian subjects, forty years old and younger, and not taking any drug impacting their periodontal health. The exclusion criteria of the present investigation included professional teeth cleaning, periodontal treatment, and acute gingival infection within 3 months of starting this trial. A biodata sheet was filled in by all included subjects. One trained specialist periodontist carried out all periodontal measurements and OPG imaging (NK). Apart from the wisdom teeth, all other teeth available were examined using the Florida probe system (Florida Probe Corporation, Gainesville, FL, USA). An orthopantomogram (OPG) was employed to report all hard tissue-related data. The Veraview X800 software program (J. MORITA MFG. CORP., Kyoto, Japan) was used to calculate the bone loss in millimeters. The periodontal clinical data included were bleeding upon probing (BoP), suppuration (present/absent), number of missing teeth, reasons for tooth loss (periodontal/non-periodontal), plaque index (PI), bone loss/age, bone loss, vertical bone loss (present/absent), periodontal pocket depth (PD), gingival recession, and clinical attachment loss (CAL). The three bands of PD and CAL were reported [27]. In the current investigation, samples of unstimulated saliva (64 samples), cheek swabs (64 samples), and pooled subgingival plaque (512) samples were taken from each participant. The 2017 classification of periodontal and peri-implant diseases and conditions was used to identify subjects with periodontitis [28].

### 2.2. PCR Detection of SEROTYPES

For each harvested sample, DNA extraction was performed using a GXT NA Extraction Kit^®®^ (Hain Lifescience, GmBH, Nehren, Germany) and an Arrow automated extraction instrument (Liaison IXT, DiaSorin Ltd., Dublin, Ireland), following the same steps included in other publications [27,29]. The quantification process of isolated DNA was performed using the NanoDrop (Thermo Fisher, Waltham, MA, USA) tool. The quantification process was performed by creating standard curves of the reference strain HK1651 of *A. actinomycetemcomitans* [29]. The qPCR was employed to quantify the total amount of *A. actinomycetemcomitans* using a Corbett Research Rotor-Gene 6000 Rotary Analyze instrument (QIAGEN, Valencia, CA, USA). The Kirakodu protocol was followed in terms of cycling requirements [30]. The primers employed were Forward (5′-CTAGGTATTGCGAAACAATTTG-3′) and Reverse (5′-CCTGAAATTAAGCTGGTAATC-3′). An amount of 100 *A. actinomycetemcomitans* cells per ml of sample was defined as a positive result. *A. actinomycetemcomitans* containing samples were used to identify the six serotypes under investigation. The Kaplan protocol was followed to identify serotypes b and c. [31]. Table 1, Table 2 and Table 3 reveal the primer information, PCR reaction set up, and PCR amplification protocol.

### 2.3. Statistical Analysis

The gathered data was assessed using IBM SPSS Statistics software program version 29.0 (SPSS Inc., Chicago, IL, USA). The target variable in the present study was the presence of *A. actinomycetemcomitans*. Descriptive statistical analysis was used to illustrate the presence of different *A. actinomycetemcomitans* serotypes. The prevalence of each serotype was calculated. Furthermore, a chi-square test was carried out to assess the relationship between *A. actinomycetemcomitans serotypes* and the grade of periodontitis, the stage of periodontitis, the extent of periodontitis, gender, age, origin, marital status, dental visits, smoking, occupation, residence, education level, family history of periodontitis, and oral hygiene method. The confidence level was set at 95% and the significance level used was 5%.

### 2.4. Ethical Considerations

The University of Western Australia granted ethical clearance to carry out the current investigation (2022/ET000252).

## 3. Results

**Figure 1:**
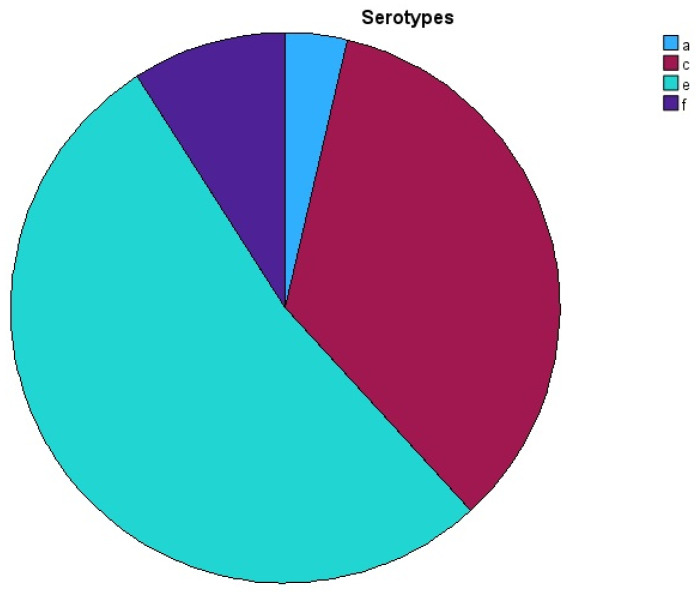
The distribution of *A. actinomycetemcomitans* serotypes a, c, e, and f.

A total of sixty-four participants were included in the current trial; among these, 37 (57.80%) were females. The mean age was 35 ± 4.70 (SD). In total, 60.90% of the patients had generalized periodontitis. Twelve patients were positive for *A. actinomycetemcomitans*; of them, seven were females and five were males. Ten of the twelve positive patients were non-Australians (China, Italy, Libya, Britain, Poland, and New Zealand). The distribution of periodontitis grades A through C was grade A (3.10%), grade B (60.90%), and grade C (35.90%). The occurrence of the periodontitis stages was stage II (7.80%), stage III (59.40%), and stage IV (32.80%). Table 4 and Figure 1 demonstrate the distribution of serotypes among patients and samples. Table 5 reveals *A. actinomycetemcomitans*-positive patients and their characteristics. The most common serotype of *A. actinomycetemcomitans* in this group was serotype e (positive in 29 samples in 12 patients, 80.55%). Serotype e was present equally in the unstimulated saliva samples and pooled subgingival plaque samples. The presence of serotype e in the cheek swabs was the lowest. The presence of serotype e in unstimulated saliva was positive in 12 samples taken from twelve patients. Serotype e isolated from the pooled subgingival plaque was positive in 12 samples taken from the same twelve patients in the unstimulated saliva sample group. Finally, the presence of serotype e in the cheek swab samples was positive in five patients from five samples only. The second most common serotype of *A. actinomycetemcomitans* in this cohort was serotype c. Serotype c was found in eight patients and nineteen samples (52.77%). Serotype c was isolated from the unstimulated saliva of six patients and six samples. In the cheek swabs, serotype c was again found in six patients and six samples, three of which were from the same group as the unstimulated saliva sample group, and the other three patients were new. In pooled subgingival plaque samples, serotype c was detected in seven patients and seven samples. Six of these patients were already positive for this serotype from the unstimulated saliva and cheek swab samples. Serotype f of *A. actinomycetemcomitans* was less frequent than serotypes e and c. Serotype f was isolated from three patients and five samples. Serotype f was found in the unstimulated saliva samples of three patients with three samples. Serotype f was isolated in one patient with one cheek swab sample. Serotype f was found in one patient with one pooled subgingival plaque sample; this patient also had a positive cheek swab sample. Serotype a of *A. actinomycetemcomitans* was less prevalent compared to all other serotypes of this bacterium. Serotype a was isolated from one patient with two samples only. Serotype a was found in one patient with one unstimulated saliva sample. Serotype a was not found in any cheek swab samples. Serotype a was found in one patient with one pooled subgingival plaque sample. This patient also had a positive unstimulated saliva sample. Serotypes b and d of *A. actinomycetemcomitans* were completely absent in this cohort and in all the samples taken. Using a chi-square test, the presence of serotype e was associated with those who lived in rural areas (*p* = 0.003) and those with low education (*p* = 0.041).

Some of the serotypes of *A. actinomycetemcomitans* existed in isolation and were not combined with another serotype. Serotype e was detected alone in nine samples from six patients (five unstimulated saliva samples, one sample from a cheek swab, and three samples from pooled subgingival plaque). Serotype c was isolated alone from three cheek swab samples from three patients. Some of the *A. actinomycetemcomitans* serotypes existed in a paired manner, like serotypes e/c, e/f, and e/a. It was always serotype e that existed with the other serotypes, but the other serotypes did not present without serotype e. Serotype e presented with serotype c in thirteen samples taken from eight patients. One of the patients had a combination of serotypes e and c in all three different samples (unstimulated saliva, cheek swab, and pooled subgingival plaque). Serotype e was presented with serotype f in three samples in the same patient. There was one patient who had serotype e of this bacterium presented alongside serotype a; this sample was taken from pooled subgingival plaque. Only three patients with three samples presented with three serotypes together in this cohort. One patient and one sample were taken from the unstimulated saliva presented as serotypes a, c, and e. Two patients presented with two samples from unstimulated saliva with serotypes e, c, and f.

## 4. Discussion

The presence of *A. actinomycetemcomitans* serotypes was investigated in twelve positive subjects in the present investigation. Serotype e was the most common serotype (80.55%) in this cohort. The second most common serotype present in this trial was serotype c (52.77%). Serotypes f and a were the lowest in terms of their presence in this study (15.62% and 6.25%). A study by Kim compared the presence of *A. actinomycetemcomitans* serotypes in one hundred and ninety-four patients diagnosed with periodontitis [15]. Forty-five samples were included in the serotype analysis in subjects from Germany; the occurrence of serotypes was as follows: b (33.30%), c (25.0%), and a (20.80%). This was compared to Korean subjects, the serotype occurrence in whom was as follows: c (61.90%) and d (19.0%). In Thailand, a study found the following serotype occurrence: c (57.0%), a (33.0%), and b (7.0%) [25]. Subgingival plaque samples from 453 subjects were assessed for *A. actinomycetemcomitans* serotypes in 86 positive subjects. These outcomes were in agreement with previous observations suggesting that Asian populations are commonly colonized with *A. actinomycetemcomitans* serotype c and occasionally infected with serotype b [15,32,33]. In contrast, serotype b was frequently observed in Caucasian populations; in the present study, we did not detect any serotype b in the selected population despite including a number of Caucasian subjects of European descent [15,34,35]. Serotypes d and f are almost undetected in most countries worldwide [22,36,37]. This finding is in accordance with the current investigation, where serotype d was completely absent. However, a high prevalence of serotype e (19–47%) was noted in Indonesian and Japanese individuals [33,38]. The outcomes from the present and other investigations indicate that different ethnic groups are affected by different *A. actinomycetemcomitans* serotypes. In the present study, individuals of Japanese and Indonesian heritages were not included; however, serotype e was the most prevalent serotype in this study. The data collected from the three different intra-oral sites were analyzed. We found that more *A. actinomycetemcomitans* serotypes were isolated from unstimulated saliva and subgingival plaque compared to cheek swabs. Biologically unstimulated saliva sampling might be a promising method of microbial diagnosis in periodontitis, as this fluid can be easily, repetitively, and non-invasively collected very quickly in a chairside manner.

Distinct evolutionary lineages of *A. actinomycetemcomitans* are discernible, and the six currently recognized serotypes constitute genetically isolated subpopulations [39]. Most patients carry a single serotype, which remains stable for a long time [22]. Occasionally, individuals are colonized with two or three serotypes, as in the present study, where up to 55.55% of the samples presented with combined serotypes. This is similar to a study in Japan, where two or three serotypes of *A. actinomycetemcomitans* were detected in 33% of the sites that tested positive for this bacterium [33]. There is evidence of differences in serotype presence related to geography and/or ethnic group. Among isolates of *A. actinomycetemcomitans* from Finland, Sweden, and Denmark, the serotypes a through c are usually positive [22,40,41]. In contrast, some research reveals a dominant occurrence of serotype c in Japanese [23,42] and Chinese populations [32,33]. The current investigation revealed domination of serotype e, followed by c. Domination of serotype e was present in Japanese patients [19]. Investigations published in the US and Scotland [43] reveal domination of serotypes a and b [44,45]. Our own data are in accordance with the literature with regard to the high occurrence of serotype c for East Asian countries; however, we are the first *A. actinomycetemcomitans* serotype study in the Oceania region [32,42,46]. In the current investigation, multiple serotypes were detected for *A. actinomycetemcomitans*-positive subjects. The high presence of multiple serotypes in these particular ethnic groups may be partly explained by the domination of serotype e, as 80.55% of the samples carrying multiple serotypes were infected by this serotype. We used PCR, which is more sensitive than serological methods, to identify *A. actinomycetemcomitans* serotypes. In addition, subgingival plaque was pooled from a large number of periodontal pockets around the examined teeth to minimize any mistakes during the sampling process. Therefore, multiple serotypes are a common occurrence in the present investigation.

In the current investigation, only 12.5% of the samples were negative for any of the serotypes assessed. In the Bandhaya study, and despite using PCR technology, 3.5% of the participants were negative for any of the serotypes under investigation [25]. These negative serotype results range from 2% to 9% and are more common with those investigations that use serological means to study serotype occurrence [22,32]. In addition, non-serotypeable strains, as detected by serological methods, may represent variants with changing expressions of specific serotype polysaccharide antigens. These variants could be assigned to the six known serotypes using the PCR method.

The link between *A. actinomycetemcomitans* serotypes and periodontitis is unclear. Research from the US has revealed that serotype b dominates subjects diagnosed with aggressive periodontitis [8]. The same outcome was detected in Finland, where serotype b commonly occurs in patients with aggressive periodontitis, whereas serotype c is associated with healthy periodontal tissue [35,47]. However, a US investigation revealed that the presence of *A. actinomycetemcomitans*, but not a specific serotype, is related to the initiation of aggressive periodontitis [48]. This was confirmed by similar investigations in Asia that revealed no significant relationship between serotypes and the extent or severity of periodontitis [38,49]. Using a chi-square test, the presence of serotype e was associated with those who lived in rural areas and those with low education; however, these findings could not be compared to other studies due to a lack of published data. The relationship between serotype b and aggressive periodontitis, particularly in African American populations, may indicate virulence differences within this serotype or differences in susceptibility among different populations. In the current investigation, and despite including both young and aged subjects diagnosed with an advanced form of periodontitis, there was no presence of serotype b of *A. actinomycetemcomitans*. However, it seems in the current investigation that serotype e of *A. actinomycetemcomitans* is associated with severe periodontitis. None of the *A. actinomycetemcomitans*-positive patients in this investigation had the JP2 genotype.

## 5. Conclusions

In patients diagnosed with severe periodontitis, serotype e was dominant. Serotypes b and d did not appear in this population. Most subjects harbor a single serotype, but some individuals were colonized with two or three serotypes.

## Figures and Tables

**Table 1:** PCR detection of serotypes of *A. actinomycetemcomitans.*

Primer Name	Primer Sequence 5′→3′	Size
Serotype b and c: forward	ARAAYTTYTCWTCGGGAATG (R = A/G; Y = C/T; W = A/T)	333 bp
Serotype b: reverse	TCTCCACCATTTTTGAGTGG	268 bp
Serotype c: reverse	GAAACCACTTCTATTTCTCC	
Serotype a: forward	GGACAAAGTGGTGTTGTTTGG	362 bp
Serotype a: reverse	GCAAGCCAACTTCTACACAATG	
Serotype e: forward	CCTTCGACCAAACGGTAAAA	283 bp
Serotype e: reverse	TTAAAAATAGCGTGCGTGAGC	
Serotype d: forward	TCCCAGAGGTTGGTTATTTTT	300 bp
Serotype d: reverse	TTCTTTCCCAAAAACCAAGTTTA	
Serotype f: forward	TTGATTTTGCAGAGGTCAATG	250 bp
Serotype f: reverse	TGGCAGAGAGTTTTCACTTGC	
Primers Annealing temperature.: 55 °C		

**Table 2:** PCR reaction set up for the detection of serotypes of *A. actinomycetemcomitans.*

Components	Volume/Reaction
2x QIAGEN Multiplex PCR Master Mix	12.5 µL
Forward and reverse primers	5 µL
5x Q-Solution	5 µL
DNA	2.5 µL
Total volume	25 µL

**Table 3:** PCR amplification protocol for the detection of serotypes of *A. actinomycetemcomitans.*

Step	Time	Temperature
Initial heat activation	15 min	95 °C
3-step cycling: denaturation	30 s	94 °C
Annealing	90 s	55 °C
Extension	60 s	72 °C
Number of cycles	40 Cycles	
Final extension	30 s	72 °C

**Table 4:** All 36 samples of *A. actinomycetemcomitans* serotypes a, b, c, d, e, and f detected.

No.	ID	Serotype	No.	ID	Serotype	No.	ID	Serotype
1	1S	c, e	13	1C	c, e	25	1P	c, e
2	7S	a, c, e	14	7C	-	26	7P	a, e
3	8S	c, e	15	8C	e	27	8P	c, e
4	13S	c, e	16	13C	c	28	13P	c, e
5	21S	e, f	17	21C	e, f	29	21P	e, f
6	22S	e	18	22C	-	30	22P	c, e
7	24S	e	19	24C	c	31	24P	e
8	27S	e	20	27C	c	32	27P	c, e
9	30S	e	21	30C	-	33	30P	e
10	31S	e	22	31C	-	34	31P	e
11	37S	c, e, f	23	37C	c, e	35	37P	c, e
12	44S	c, e, f	24	44C	c, e	36	44P	c, e

S: saliva sample; C: cheek swab sample; P: polled subgingival plaque sample; -: negative result.

**Table 5:** *A. actinomycetemcomitans*-positive patients and their characteristics.

Patient/Variables	Gender	Age in Years	Country of Origin	Stage/Grade
Pt 1	M	40	China	IV/C
Pt 2	F	38	Australia	II/B
Pt 3	M	38	New Zealand (Māori)	III/B
Pt 4	M	33	Vietnam	IV/C
Pt 5	F	36	Libya	III/B
Pt 6	M	40	Australia	III/B
Pt 7	F	39	Poland	III/B
Pt 8	F	39	Australia	III/B
Pt 9	F	32	New Zealand (Māori)	IV/C
Pt 10	F	36	Italy	IV/C
Pt 11	M	38	New Zealand	III/B
Pt 12	F	32	Italy	III/B

Pt: patient, M: male, F: female.

## Data Availability

The raw data supporting the conclusions of this study will be made available by the authors.
